# Ovarian response prediction in controlled ovarian stimulation for IVF using anti-Müllerian hormone in Chinese women: A retrospective cohort study

**DOI:** 10.1097/MD.0000000000006495

**Published:** 2017-03-31

**Authors:** Haiyan Zheng, Shiping Chen, Hongzi Du, Jiawei Ling, Yixuan Wu, Haiying Liu, Jianqiao Liu

**Affiliations:** Center for Reproductive Medicine, Key Laboratory for Reproductive Medicine of Guangdong Province, Key Laboratory for Major Obstetric Diseases of Guangdong Province, Key Laboratory of Reproduction and Genetics of Guangdong Higher Education Institutes, Third Affiliated Hospital of Guangzhou Medical University, Guangzhou, Republic of China.

**Keywords:** anti-Müllerian hormone, Chinese women, controlled ovarian stimulation, ovarian response

## Abstract

The predictive value of anti-Müllerian hormone (AMH) in Chinese women undergoing in vitro fertilization (IVF) treatment is data deficient. To determine the attributes of AMH in IVF, oocyte yield, cycle cancellation, and pregnancy outcomes were analyzed. All patients initiating their first IVF cycle with gonadotropin-releasing hormone agonist treatment in our center from October 2013 through December 2014 were included, except patients diagnosed with polycystic ovarian syndrome. Serum samples collected prior to IVF treatment were used to determine serum AMH levels. A total of 4017 continuous cycles were analyzed. The AMH level was positively correlated with the number of oocytes retrieved. Overall, AMH was significantly correlated with risk of cycle cancellation, poor ovarian response (POR, 3, or fewer oocytes retrieved) and high response (>15 oocytes), with an area under the curve (AUC) of 0.83, 0.89, and 0.82 respectively. An AMH cutoff of 0.6 ng/mL had a sensitivity of 54.0% and a specificity of 90.0% for the prediction of cycle cancellation, and cutoff of 0.8 ng/mL with a sensitivity of 55.0% and a specificity of 94.0% for the prediction of POR. Compared with AMH >2.0 ng/mL, patients with AMH < 0.6 ng/mL had a 53.6-fold increased risk of cancellation (*P *< 0.001), and AMH <0.80 ng/mL were 17.5 times more likely to experience POR (*P* < 0.001). However, AMH was less predictive of pregnancy and live birth, with AUCs of 0.55 and 0.53, respectively. Clinical pregnancy rate, ongoing pregnancy rate, and live birth rate per retrieval according to the AMH level (≤0.40, 0.41–0.60, 0.61–0.80, 0.81–1.00, 1.01–1.50, 1.51–2.00, and >2.00 ng/mL) showed no significant differences. Even with AMH≤0.4 ng/mL, 50.0% of all the patients achieved pregnancy and 34.8% of patients achieved live birth after transfer. Our results suggested that AMH is a fairly robust metric for the prediction of cycle cancellation and oocyte yield for Chinese women, but it is a relatively poor test for prediction of pregnancy outcomes. Patients with low levels of AMH still can achieve reasonable treatment outcomes and low AMH levels in isolation do not represent an appropriate marker for withholding fertility treatment.

## Abstract 2

Supplemental Digital Content is available in the text

## 1 Introduction

The optimization and individualization of controlled ovarian stimulation (COS) for in vitro fertilization (IVF) have become increasingly important. The correct individualization of the gonadotrophin start dose is an extremely important clinical decision for the ideal treatment protocol, which is based on the correct prediction of ovarian response. Evaluation of the ovarian reserve is necessary to achieve an appropriate COS. The term ovarian reserve usually refers to the size of the primordial follicle pool as well as the oocyte quality.^[1,2]^ There are now several modalities to measure ovarian reserve, including anti-Müllerian hormone (AMH), day-3 follicle-stimulating hormone (FSH), and antral follicle count (AFC), each with its own merits and shortcomings. The basal FSH level is influenced by the menstrual cycle and has limited use for predicting poor and high responders. Ultrasonographic markers, such as AFC and ovarian volumes, have been shown to be affected by inter observer variation.^[3–5]^

As serum AMH levels reflect the primordial follicle pool indirectly, this hormone seems to be a promising biochemical marker for the detection of decreased ovarian reserve as early as possible. It has been demonstrated that AMH is an accurate predictor of both high^[6]^ and low ovarian response in either gonadotropin-releasing hormone (GnRH) agonist treatment or GnRH antagonist treatment,^[7,8]^ suggesting it would be an ideal marker for the individualization of COS strategies. A recent prospective study indicated that AMH and basal FSH are statistically significant predictors of both the number of oocytes retrieved and the occurrence of an excessive ovarian response, whereas AMH alone was the main predictor for low ovarian response.^[9]^ Indeed, the use of an AMH-tailored approach has previously been suggested by several investigators.^[10–12]^ Up to date, several cutoff points have been proposed for AMH in order to predict ovarian response, but a consensus cannot be achieved due to the different COS protocols or different populations. Besides, AFC has been reported to possess similar performance as AMH in predicting the number of oocytes retrieved in previous studies in IVF patients treated with GnRH agonist protocols.^[13–16]^

Although acceptance is fairly universal that AMH is correlated with response to stimulation, it remains more controversial how, if at all, AMH is correlated with IVF outcomes. Moreover, AMH detection has not been widely used in clinical work in China, there is a dearth of literature regarding the predictive value of AMH in Chinese women undergoing IVF treatment, with much of the data reported coming from patients in Europe and United States.^[17]^ To investigate the value of AMH compared with that of women age and FSH as predictors of ovarian response in Chinese women undergoing a first cycle of COS with exogenous gonadotropins, we set out to analyze our own experience with the second-generation AMH assay since we implemented it as a routine part of our center's infertility workup in June 2013.

## 2 Methods

### 2.1 Cycle inclusion criteria

This study was approved by the Third Affiliated Hospital of Guangzhou Medical University institutional review board. Our center began using the second-generation AMH assay as an element of the fertility workup in June 2013. All IVF cycles at the Center for Reproductive Medicine of Affiliated Hospital of Guangzhou Medical University from October 2013 through December 2014 were analyzed for potential inclusion. Inclusion criteria were all women who had an AMH level assessed within the previous 12 months before their IVF cycle start. None of the women included were using hormonal contraception immediately before AMH determination. The selection was limited to patients with a regular cycle who underwent their first IVF/ intracytoplasmic sperm injection (ICSI) cycle with GnRH agonist treatment. Women diagnosed with polycystic ovary syndrome (PCOS) were excluded in the present study.

### 2.2 AMH assay

After blood collection, serum for assay of AMH was separated and AMH levels were determined. All measurements were performed in a batch analysis using a DS2 ELISA robot with a single lot reagent (AMH Gen II ELISA, Beckman Coulter; Inc.). The lowest and highest detectable level of AMH was 0.09 ng/mL and 16.0 ng/mL, respectively. However, because of the infrequency of samples found in this range, all low levels that were <0.09 ng/mL (n = 4) were reported as such. AMH levels > 16.0 ng/mL was calculated according to the testing curve. Interassay variability was 9.8% and 6.5% for an AMH level of 0.35 ng/mL and 4.0 ng/mL, respectively.

### 2.3 Stimulation regimens

A standard long protocol was used for controlled ovarian stimulation. The GnRH-analog (Triptorelin acetate; Ipsen Pharma Biotech, France) was administered at a dose of 1.0 mg intramuscularly in the midluteal phase of the preceding menstrual cycle, or approximately 7 days before menstruation. Ovarian stimulation was effected with exogenous gonadotrophins in the form of recombinant FSH (Gonal-f, Merck Serono, Germany), or Urofollitropin for Injection (Lishenbao, Livzon Pharmaceutical Group Inc., China). The starting daily dose was decided according to age, AMH levels, antral follicle count, and baseline FSH levels. In all cases, ovarian stimulation was carried out to maximize follicular response while minimizing risk of ovarian hyperstimulation syndrome (OHSS). Serum estradiol (E_2_), luteinizing hormone (LH), and progesterone (P) concentrations were measured as well as transvaginal ultrasound scan was arranged on days 8 and 10 of ovarian stimulation and every 1 or 2 days thereafter, as required. Patients with 3 or fewer follicles were counseled regarding the risks and benefits of continuing their IVF cycle versus cancellation. In general, patients with 3 or more follicles were encouraged to proceed with IVF.

Final oocyte maturation was induced with recombined-human chorionic gonadotropin (hCG, Ovitrelle, Merck Serono, Germany); or 6000 IU to 10,000 IU of Chorionic Gonadotrophin for Injection (Livzon Pharmaceutical Group Inc., China), provided that there was at least 2 leading follicles attained a mean diameter of 17 mm. Transvaginal ultrasound-guided oocyte retrieval was undertaken 34 to 36 hours after hCG injection and embryo transfer was performed 3 days later. A dose of 40 mg intramuscular progesterone was used to support the luteal phase until the day of urine pregnancy test (14 days post embryo transfer).

### 2.4 Outcome measures

The primary outcome measure was correlation analysis between ovarian response and AMH levels. A high response was arbitrarily defined as >15 oocytes retrieved.^[6]^ A poor response was defined as<4 retrieved oocytes or cancellation due to low ovarian response (≤3 dominant follicles <12 mm diameter).^[7]^ A normal response was therefore defined as 4 to 15 oocytes retrieved. The duration of stimulation, total cumulative dose of gonadotropins, total number of oocytes retrieved, risk of cycle cancellation, clinical and ongoing pregnancy were the secondary outcome variables assessed.

The clinical pregnancy rate was defined as the number of cycles with at least 1 fetal heartbeat at 6 weeks’ gestation divided by number of cycles with transfer. The ongoing pregnancy rate was defined as the number of live births plus number of ongoing gestations at 24 or more weeks’ gestation.

### 2.5 Statistical analysis

Statistical analysis was carried out using the Statistical Program for Social Sciences (SPSS Inc., Version 20.0, Chicago). Data for continuous variables are presented as mean values and standard deviation. Between-group statistical comparisons of the mean values were performed with analysis of variance tests. Χ^2^ tests were used for categorical data. Spearman or Pearson correlation coefficients (*r*) were calculated to evaluate the relationships between continuous variables (e.g., number of oocytes retrieved and AMH level), depending on whether data were normally distributed. Receiver operating characteristic (ROC) curves were generated for AMH, women age, and FSH to compare ability of parameters to predict poor or high ovarian response. The sensitivity and specificity values were calculated for selected AMH cutoff levels. Logistic regression was performed to assess the effect of AMH levels on binary outcomes. The capability of AMH, age, and day3 FSH to predict the number of oocytes retrieved was evaluated using a stepwise forward selection procedure within an analysis of covariance model framework. The procedure sequentially selected the predictor variables according to the increase in the coefficient of determination (*R*^2^). All *P* values were based on 2-sided tests and *P* < 0.05 was considered to be statistically significant.

## 3 Results

From October 2013 through December 2014, 4017 individual patients undergoing their first IVF cycle had an AMH level drawn at our center. So as to minimize repeated measures bias, data presented henceforth will be limited to first cycles only.

### 3.1 Patient demographics and stimulation characteristics

Twenty-four cycles were cancelled due to poor ovarian response. Then, 3983 retrieval cycles remained were divided into 3 subgroups according to the ovarian response category: high (n = 1190), normal (n = 2609), and low (n = 184). Demographics, baseline characteristics, and main outcome parameters of all the retrieval cycles are presented in Supplemental Digital Content (see Table S1 Supplemental Content, which illustrates the baseline and stimulation characteristics by subgroup for women undergoing IVF/ICSI). Among 3983 patients, 3075 (77.2%) were scheduled to undergo an IVF treatment, with ICSI to be performed in 821 (20.6%) patients, and 87 (2.2%) were performed with IVF+ICSI. There was a significant between-group difference for the age, day3 FSH, AMH level, total dose of gonadotropin, number of oocytes retrieved, number of embryos available, clinical pregnancy, ongoing pregnancy, and live birth (*P* < 0.05).

### 3.2 Predictive value of AMH in ovarian response

**Figure 1:**
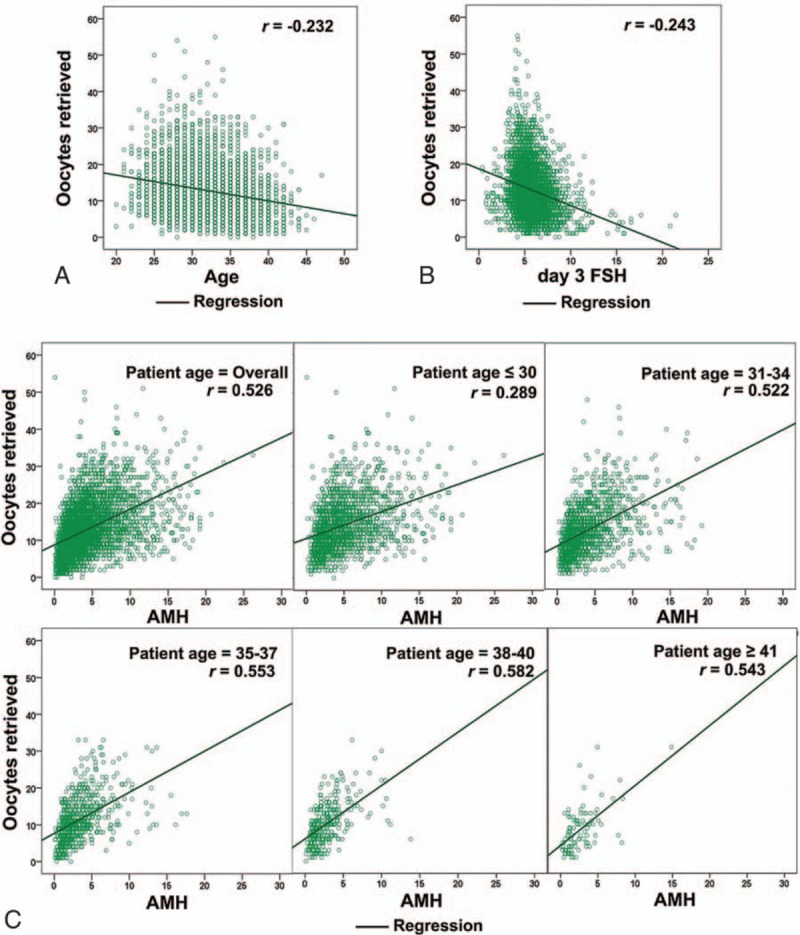
Correlation analysis between ovarian reserve makers and oocytes yield. (A) Correlation between the age and the number of oocytes retrieved. (B) Correlation between day 3 FSH and the number of oocytes retrieved. (C) Correlation between AMH and the number of oocytes retrieved (stratified by the age). The correlation coefficients (*r*) were calculated to evaluate the relationships between continuous variables and shown in the top right. AMH = anti-Müllerian hormone, FSH = follicle-stimulating hormone.

Overall, AMH was a markedly better predictor of the number of oocytes retrieved than age and day3 FSH in our study cohort. As shown in Fig. 1, a positive linear correlation exists between increasing AMH values and total number of oocytes retrieved (*r* = 0.526 overall, *P* < 0.001), and a negative linear correlation between age, day3 FSH, and oocytes number (*r* = –0.232 for age, *r* = –0.243 for FSH, *P* < 0.001), respectively. For each age group, especially for patients ≧ 30 years old, the number of total eggs retrieved increased significantly with increasing AMH value (*r* range, 0.522–0.582, all *P* < 0.001). The stepwise forward procedure for prediction of number of oocytes retrieved provided similar findings on the contribution of the selected predictors for the whole cohort (Table 1). AMH was identified as the single variable with the highest coefficient of determination: *R*^2^ = 0.213. The *R*^2^ for age and day3 FSH was only 0.049 and 0.058, respectively, and inclusion of age or FSH or both together in the models had no significant improvement on the prediction of oocyte yield.

**Table 1:**
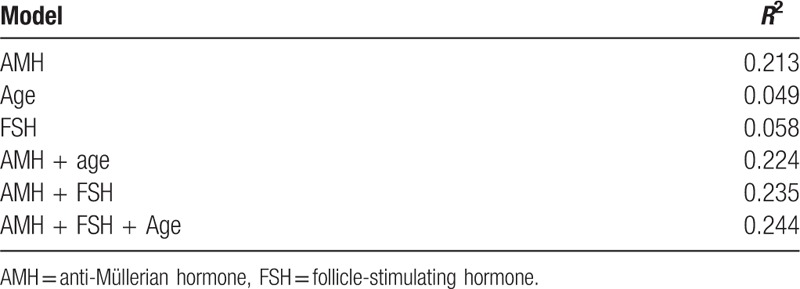
Stepwise analysis of covariance models in patients undergoing controlled ovarian stimulation with gonadotropin-releasing hormone agonist protocol.

**Figure 2:**
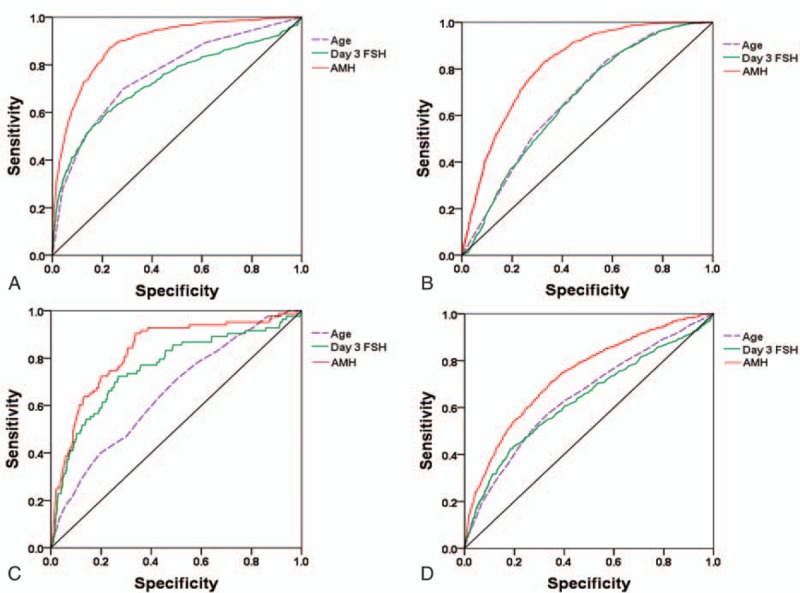
Receiver operating characteristic curves for prediction of ovarian response. (A) Low response (≤3 oocytes, AUC = 0.89). (B) High response (≥15 oocytes, AUC = 0.82). (C) Cycle cancellation (AUC = 0.83). (D) No embryo available (AUC = 0.74). AUC = area under the curve.

ROC curves were plotted for several predictors included AMH, age, and day3 FSH. The levels of accuracy, as expressed by the AUCs, for ovarian response prediction are depicted in Table 2. AMH exhibited an area under the curve (AUC) of 0.83, 0.89, and 0.82 for the ability to predict cycle cancellation, low response (≤3 oocytes), and high response (≥15 oocytes), respectively (Fig. 2A–C). The clinical value of different AMH cut-offs for ovarian response prediction was illustrated in detail (Table 2). An AMH cutoff of < 0.40 ng/mL had a sensitivity of 39.0% and specificity of 94.0% for predicting cycle cancellation due to ovarian poor response. Alternatively, an AMH cutoff of 0.6 ng/mL had a sensitivity of 54.0% and a specificity of 90.0% for the prediction of cycle cancellation. The performance of AMH as a test for the prediction of low response was limited, as reflected by the low sensitivities corresponding with lower AMH thresholds. The optimal cut-off point seems to lie at a level of 0.80 ng/mL, thus identifying 55% of all low responders.

**Table 2:**
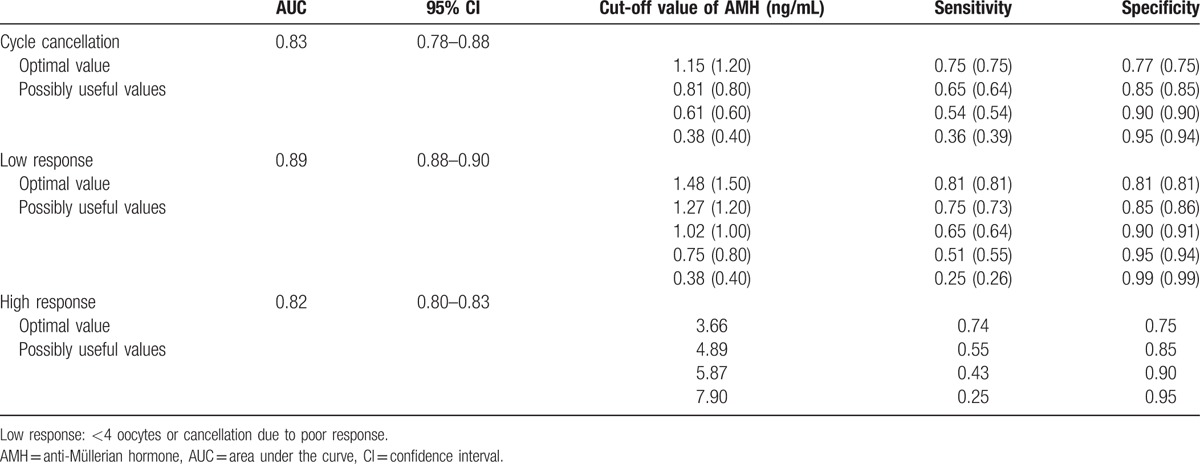
Test characteristics for anti-Müllerian hormone as a predictor of the outcome of cycle cancellation, low and high response.

Furthermore, Table 3 shows the quantified overall increased risk (via logistic regression) of cycle cancellation and poor ovarian response with decreasing AMH. Patients with AMH ≤0.60 ng/mL were 53.6 times more likely than patients with AMH >2.0 ng/mL to be cancelled (95% confidence interval [CI], 15.1–190.3; *P* <0.001). At the same time, patients with AMH ≤0.80 ng/mL were 17.5 times more likely to be shown as poor ovarian response than patients with AMH >2.0 ng/mL (95% CI, 9.8–31.4; *P* < 0.001). Exactly, the cycle cancellation rate and the poor ovarian response rate increased dramatically to 8.3% and 25.0% when AMH decreased to 0.60 ng/mL and 0.80 ng/mL, respectively (Table 4). The same results can also be achieved when the cycle cancellation rate was compared according to the AMH level stratified by the patient age (see Table S2, Supplemental Content which illustrates the incidence of cycle cancellation according to anti-Müllerian hormone and age).

**Table 3:**
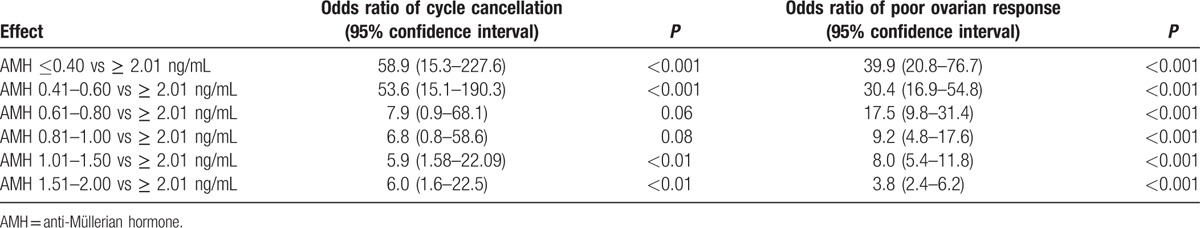
Odds ratios of cycle cancellation and poor ovarian response according to the anti-Müllerian hormone level.

**Table 4:**
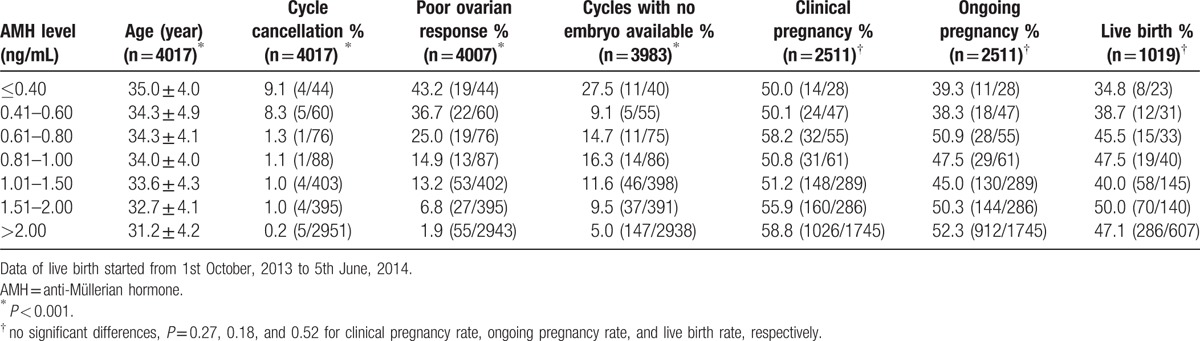
Comparison of ovarian response and clinical outcome according to anti-Müllerian hormone level.

For predicting high response, when choosing a higher test cut-off level, the sensitivity decreased, whereas the specificity increased (Table 2). At a specificity level of 85% and test cut-off of 4.89 ng/mL, the test seemed to have the best performance level, indicating that in the case of an abnormal test result, the chance of having an excessive response is 55%.

### 3.3 Predictive value of AMH in clinical outcomes

Clinical outcomes were next analyzed with respect to serum AMH levels. AMH exhibited an AUC of 0.74 (95% CI, 0.72–0.76) for the ability to predict cycles with no embryo available (Fig. 2D). An AMH cutoff of < 1.20 ng/mL had a sensitivity of 55.0% and specificity of 80.0% for predicting cycles with no embryo available (data not shown).

However, AMH was less predictive of pregnancy and live birth, with AUCs of 0.55 (95%CI, 0.53–0.57) and 0.53 (95%CI, 0.50–0.57), respectively (data not shown). Clinical pregnancy rate (CPR), ongoing pregnancy rate (OPR), and live birth rate (LBR) per retrieval according to AMH level (≤0.40, 0.41–0.60, 0.61–0.80, 0.81–1.00, 1.01–1.50, 1.51–2.00, and >2.00 ng/mL) showed no significant differences (*P* = 0.27, 0.18, and 0.52 for CPR, OPR, and LBR, respectively, Table 4). Even with AMH≤0.4 ng/mL, 50.0% of all the patients achieved pregnancy and 34.8% of patients achieved live birth after transfer. In addition, Table 5 showed the ongoing clinical pregnancy rate per retrieval according to the AMH level, stratified by the patient age (≤30, 31–34, 35–37, 38–40, 41–42, and >40 years). For each age group, there was no statistically significant difference in ongoing pregnancy rates with increasing AMH.

**Table 5:**
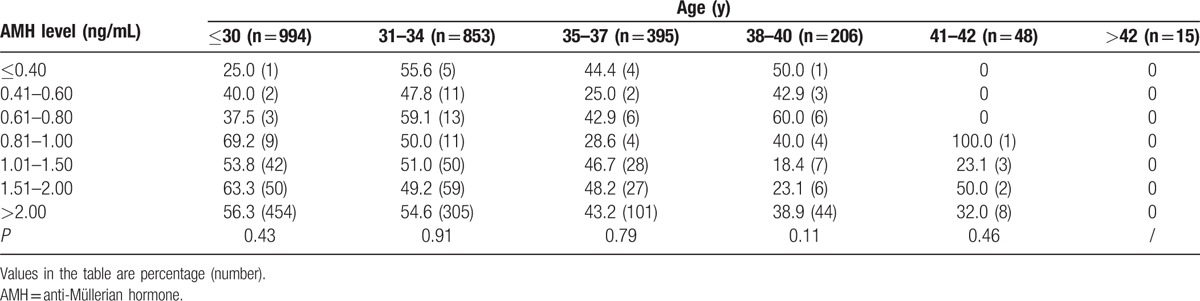
Ongoing clinical pregnancy per retrieval according to anti-Müllerian hormone and age.

## 4 Discussion

Despite many advances in the field of human assisted reproduction, the risk of extremes of ovarian response following COS is still a considerable problem in many programs. For most fertility physicians in China, in addition to the experience from their own clinical practice, they largely rely on the woman's age, the presence or absence of polycystic ovary (PCO) appearance, and the basal FSH levels to determine the starting dose of gonadotrophins for stimulation. Recent years, AMH, a new ovarian reserve maker, has increasingly become a mainstay of the fertility workup in many countries since its relationship to ovarian response was first noted.^[18]^ However, the predictive value of AMH in Chinese women undergoing IVF treatment is data deficient. In the present study, we analyzed the association between AMH and a number of outcomes associated with IVF, including the number of oocytes retrieved (poor ovarian response, normal response, and high response), cycle cancellation, pregnancy, ongoing pregnancy, and live birth. Meanwhile, the predictive value of AMH in ovarian response was compared with women's age and day3 FSH. This retrospective cohort study demonstrates that AMH as a single test has substantial accuracy in the prediction of ovarian response using GnRH agonist down-regulation for IVF but is a relatively poor test for prediction of pregnancy and live birth.

A standardized definition of poor ovarian response as the retrieval of <4 oocytes following a standard IVF protocol was recently established by the European Society of Human Reproduction and Embryology Consensus Conference.^[19]^ Prediction of poor response is great of importance for the counseling and management of infertile women in IVF clinical practice. First, the finding that AMH was a more robust biomarker of the ovarian response to gonadotropins than women's age and day3 FSH was confirmed in the present study. Inclusion of age and FSH in stepwise logistic regression models did not improve the prediction of oocyte yield. Moreover, an important factor when using ovarian reserve markers as predictors of ovarian response is to establish the most sensitive markers and acceptable cut-off levels for these markers. Previous studies showed that cut-off values of AMH for predicting poor ovarian response make a great difference from 0.1 and 2.97 ng/mL.^[20]^ Nelson et al^[21]^ has reported that the best cut-off value for AMH was 0.7 ng/mL, which has 75% sensitivity in predicting poor response with a specificity of 91%, whereas another study found that an AMH value of 1.36 ng/mL was associated with a sensitivity of 75.5% and specificity of 74.8%.^[5]^ The findings from the present study are in line with the preceding 2 studies on the predictive value of AMH for ovarian response using GnRH agonist treatment. In our study, at a specificity level of 95% and test cut-off of 0.8 ng/mL, the test seemed to have the best performance level, indicating that in the case of an abnormal test result, the chance of having a poor response is 55%. Furthermore, AMH is useful in terms of counseling patients regarding their risk of cycle cancellation, as shown by our data revealing a 54.0% cancellation rate for poor response in patients with AMH <0.6 ng/mL. According to the present study and published data, we concluded that an AMH value of about 1.0±0.3 ng/mL may be considered acceptable for the prediction of poor ovarian response in IVF. On the basis of appropriate cut-off values for AMH, the prediction of poor response becomes fairly easy and is certainly useful for counseling women especially of the possible negative IVF outcomes such as cancellation of cycle and increased treatment burden. Adequate assessment of ovarian reserve may increase women's psychological comfort during the treatment cycle and perhaps reduce the number of dropouts particularly among women with an expected poor outcome.

The ovarian hyperstimulation syndrome (OHSS) is another notable iatrogenic complication in IVF treatment, and “high response” is generally termed as the retrieval of >15^[22,23]^ oocytes following a standard COS protocol. As is known to all, women with PCOS are at high risk of OHSS. However, patients with PCOS constitute only 20% of subjects undergoing COS and less than one-fifth of those will present with symptoms of OHSS.^[24]^ Therefore, as extremes of response may occur unexpectedly, there is a real need for finding predictive factors that can be used in daily clinical practice to improve the whole patient's IVF experience and to predict the individual stimulation outcome. A number of studies have demonstrated the usefulness of AMH in refining the starting dose of gonadotropins so as to maximize response while minimizing risk of OHSS.^[10,11,25,26]^ We also confirmed the high predictive value of AMH in ovarian high response, and the ROC analysis showed the AUC as 0.82. Our data indicates that a cutoff of AMH >4.89 ng/mL would have 55% sensitivity in predicting overstimulation with a specificity of 85%. But unfortunately the incidence of OHSS cannot be analyzed due to the incomplete data, which is a drawback of this study.

The potential value of AMH in predicting the likelihood of pregnancy after assisted conception has been contentious. However, the accuracy curves in this study indicate that AMH is a robust predictor for ovarian response but not for clinical pregnancy or live birth. In the past, some authors have hold the view that AMH is associated with oocyte quality,^[27]^ but more subsequent studies demonstrated that no such correlation exists.^[28]^ Our findings reveal relatively poor ROC curves for pregnancy prediction, along with no change in live birth rates according to AMH, supporting this notion. Collectively, the levels of AMH may indicate the ovarian response, that is, the number of oocytes and embryos, but not the oocyte quality.^[29–31]^ Hence, extremely low AMH levels do not seem to represent an strong marker for withholding fertility treatment. Although AMH was a poor predictor of embryo quality and pregnancy,^[17,32]^ a positive association between AMH and cumulative live-birth rates has previously been reported in the GnRH antagonist protocol. A recent powerful meta-analysis concluded that AMH alone may have some association with predicting live birth after IVF and may be helpful when counseling couples before undergoing fertility treatment, but its predictive accuracy is poor.^[33]^ Therefore, the association potentially reflects the availability of more embryos for transfer in patients with higher AMH rather than a direct association between AMH and embryo quality.^[17]^

There are a number of limitations of this study. First, another important indicator AFC was not included in our study also due to the incomplete data. Previous studies assumed that AFC and AMH possess similar performance on ovarian reserve prediction. A recent review showed that AFC can be used to reliably predict ovarian response in IVF but there is considerable variability in agreed AFC cut-off levels used for predicting poor response, which vary between 3^[34]^ and 12.^[35]^ A possible reason for such variability is the absence of a standardized measurement of antral follicles with different studies measuring different follicle populations: 2 to 5, 2 to 9, or 5 to 9 mm. Overall, the lack of reproducibility would emphasize the necessity for individual clinics to better standardize the assessment of AFC.^[36]^ Three recent large, multicenter trials showed that AFC by itself was a poorer predictor of the ovarian response to COS than AMH, and furthermore, that AFC provided no added predictive value beyond AMH.^[9,17,32]^ However, Reichman et al^[37]^ noted that AMH should not be interpreted in isolation, but rather should be addressed in the context of AFC, patient age, day-3 FSH, and prior response to stimulation. Finally, only patients with an anticipated good prognosis to gonadotropin stimulation using long protocol were included; thus, this selection may have attenuated the overall strength of the correlations given that only women within the normal range of AFC values were examined, but this limitation would also apply to AMH.

## 5 Conclusions

Our results showed that for Chinese women, anti-Müllerian hormone is a fairly robust metric for the prediction of cancellation and how many oocytes may be retrieved after stimulation but is a relatively poor test for prediction of pregnancy and live birth. After decades of practice using IVF, it is now very clear that the “one size fits all” approach may no longer exist. The correct measurement of markers of ovarian reserve allows a scientific estimate of the pool of follicles that potentially respond to ovarian stimulation. Patients with low levels of AMH still can achieve reasonable treatment outcomes and we strongly believe that low AMH levels in isolation do not represent an appropriate marker for withholding fertility treatment.^[22,38]^ We hope that to some extent this study can provide physicians some clinical data on the application of AMH in IVF treatment for Chinese women. Still future research is needed to further define the role of AMH in IVF outcome.

## Supplementary Material

Supplemental Digital Content

## Supplementary Material

Supplemental Digital Content
