# Supplementary material for: Ovarian response prediction in controlled ovarian stimulation for IVF using anti-Müllerian hormone in Chinese women: A retrospective cohort study
Source: Medicine (Baltimore). 2017 Mar 31;96(13):e6495. doi: 10.1097/MD.0000000000006495 (PMC5380281; doi:10.1097/MD.0000000000006495)
Supplement: Supplemental Digital Content [file medi-96-e6495-s001.doc]

**Title: Ovarian response prediction in controlled ovarian stimulation for IVF using anti-müllerian hormone in Chinese women: A retrospective cohort study**

**First author: Haiyan Zheng**

**TABLE S1. Baseline and stimulation characteristics by subgroup for women undergoing in vitro fertilization/intracytoplasmic sperm injection**

|  | **High response**  **(*n* = 1190)** | **Normal response**  **(*n* = 2609)** | **Low response**  **(*n* = 184)** | ***P* value** |
| --- | --- | --- | --- | --- |
| **Demographics** | | | | |
| Age (years) | 30.6±3.9 | 32.1±4.3 | 34.8±4.5 | <.001 |
| BMI (kg/m2) | 21.2±3.4 | 22.5±4.1 | 21.0±3.7 | .53 |
| Day3 FSH (IU/L) | 5.3±1.2 | 5.8±1.6 | 6.9±2.6 | <.001 |
| AMH prior to treatment (ng/ml) | 6.1±3.6 | 3.6±2.8 | 1.7±1.5 | <.001 |
| **Fertility characteristics** | | | | |
| Primary subfertility, n (%) | 646/1190 (54.3) | 1370/2609 (52.5) | 85/184 (46.2) | .11 |
| Secondary subfertility, n (%) | 544/1190 (45.7) | 1239/2609 (47.5) | 99/184 (53.8) | .11 |
| Duration of subfertility (years) | 4.3±2.7 | 4.9±3.3 | 5.5±3.8 | <.001 |
| **Cause of subfertility, n (%)** .18 | | | | |
| Male factor | 247/1190 (20.8) | 466/2609 (17.9) | 33/184 (17.9) |  |
| Unexplained | 37/1190 (3.1) | 114/2609 (4.4) | 6/184 (3.3) |  |
| Tubal factor | 753/1190 (63.3) | 1590/2609 (60.9) | 110/184 (59.8) |  |
| Endometriosis | 29/1190 (2.4) | 98/2609 (3.8) | 15/184 (8.2) |  |
| Ovulation dysfunction | 4/1190 (0.3) | 8/2609 (0.3) | 2/184 (1.1) |  |
| Mixed or other | 120/1190 (10.1) | 333/2609 (12.8) | 18/184 (9.8) |  |
| **Stimulation characteristics** | | | | |
| Total dose of gonadotropin (IU) | 2303.8±849.4 | 2941.0±1075.3 | 3830.6±1273.4 | <.001 |
| Total duration of stimulation (days) | 12.8±2.1 | 12.9±2.0 | 12.9±2.1 | .26 |
| **Clinical outcome per started cycle** | | | | |
| Total number of oocytes | 21.2±5.3 | 9.9±3.2 | 2.2±0.8 | <.001 |
| Number of embryos available | 8.4±4.1 | 4.1±2.6 | 1.0±0.8 | <.001 |
| Clinical pregnancy, n (%) | 256/387 (66.1) | 1136/2016 (56.3) | 43/108 (39.8) | <.001 |
| Ongoing pregnancy, n (%) | 220/387 (56.8) | 1014/2016 (50.3) | 38/108 (35.2) | <.001 |
| Live birth, n (%) | 96/178 (53.9) | 360/795 (45.3) | 12/46 (26.1) | <.01 |

BMI = body mass index, FSH = follicle stimulation hormone, AMH = anti-müllerian hormone,
