# Supplementary material for: Ovarian response prediction in controlled ovarian stimulation for IVF using anti-Müllerian hormone in Chinese women: A retrospective cohort study
Source: Medicine (Baltimore). 2017 Mar 31;96(13):e6495. doi: 10.1097/MD.0000000000006495 (PMC5380281; doi:10.1097/MD.0000000000006495)
Supplement: Supplemental Digital Content [file medi-96-e6495-s002.doc]

**Title: Ovarian response prediction in controlled ovarian stimulation for IVF using anti-müllerian hormone in Chinese women: A retrospective cohort study**

**First author: Haiyan Zheng**

**TABLE S2. Incidence of cycle cancellation according to anti-müllerian hormone level and age**

| **AMH level (ng/ml)** | **Age (year)** | | | | | | |
| --- | --- | --- | --- | --- | --- | --- | --- |
|  | **≤30**  **(n =1631)** | **31-34**  **(n = 1337)** | | **35-37**  **(n = 648)** | **38-40**  **(n = 304)** | **41-42**  **(n = 73)** | **＞42**  **(n = 24)** |
| ≤0.40 | 0 | 20.0 (3) | 0 | | 0 | 50.0 (1) | 0 |
| 0.41-0.60 | 25.0 (2) | 6.9 (2) | 0 | | 12.5 (1) | 0 | 0 |
| 0.61-0.80 | 0 | 0 | 4.5 (1) | | 0 | 0 | 0 |
| 0.81-1.00 | 0 | 0 | 4.5 (1) | | 0 | 0 | 0 |
| 1.01-1.50 | 1.0 (1) | 0 | 2.1 (1) | | 1.8 (1) | 0 | 0 |
| 1.51-2.00 | 0.9 (1) | 1.3 (2) | 1.2 (1) | | 0 | 0 | 0 |
| ＞2.00 | 0.2 (3) | 1 (0.1) | 0 | | 0.6 (1) | 0 | 0 |
| *P* value | <.001 | <.001 | <.05 | | .06 | <.001 | / |

Values in the table are percentage (number); AMH = anti-müllerian hormone.
